# Telerehabilitation in patients with recent hospitalisation due to acute decompensated heart failure: protocol for the Tele-ADHF randomised controlled trial

**DOI:** 10.1186/s12872-023-03407-4

**Published:** 2023-07-29

**Authors:** Mayke M. C. J. van Leunen, Ignace L. J. de Lathauwer, Cindy C. A. G. Verstappen, Dianne M. G. Visser-Stevelink, Rutger W. M. Brouwers, Cyrille Herkert, René A. Tio, Ruud F. Spee, Yuan Lu, Hareld M. C. Kemps

**Affiliations:** 1 Department of Cardiology, Máxima Medical Centre, De Run 4600, 5504 DB Veldhoven, The Netherlands; 2 Department of Industrial Design, Eindhoven University of Technology, Eindhoven, the Netherlands; 3 Department of Cardiology, Catharina Hospital Eindhoven, Eindhoven, the Netherlands; 4 Department of Electrical Engineering, Eindhoven University of Technology, Eindhoven, the Netherlands

**Keywords:** Cardiac rehabilitation, Cardiac telerehabilitation, Home based rehabilitation, Heart failure, Acute decompensated heart failure, Frailty, Physical functional capacity, Remote patient management, Telemonitoring

## Abstract

**Background:**

Cardiac rehabilitation in patients with chronic heart failure (CHF) has favourable effects on exercise capacity, the risk at hospital (re-)admission and quality of life. Although cardiac rehabilitation is generally recommended it is still under-utilised in daily clinical practice, particularly in frail elderly patients after hospital admission, mainly due to low referral and patient-related barriers. Cardiac telerehabilitation (CTR) has the potential to partially solve these barriers. The purpose of this study is to evaluate the effects of CTR as compared to standard remote care after hospital admission on physical functional capacity in CHF patients.

**Methods:**

In this randomised controlled trial, 64 CHF patients will be recruited during hospitalisation for acute decompensated heart failure, and randomised to CTR combined with remote patient management (RPM) or RPM alone (1:1). All participants will start with RPM after hospital discharge for early detection of deterioration, and will be up titrated to optimal medical therapy before being randomised. CTR will start after randomisation and consists of an 18-week multidisciplinary programme with exercise training by physical and occupational therapists, supported by a (remote) technology-assisted dietary intervention and mental health guiding by a physiologist. The training programme consists of three centre-based and two home-based video exercise training sessions followed by weekly video coaching. The mental health and dietary programme are executed using individual and group video sessions. A wrist-worn device enables remote coaching by the physical therapist. The web application is used for promoting self-management by the following modules: 1) goal setting, 2) progress tracking, 3) education, and 4) video and chat communication. The primary outcome measure is physical functional capacity evaluated by the Short Physical Performance Battery (SPPB) score. Secondary outcome measures include frailty scoring, recovery after submaximal exercise, subjective health status, compliance and acceptance to the rehabilitation programme, and readmission rate.

**Discussion:**

The Tele-ADHF trial is the first prospective randomised controlled trial designed for evaluating the effects of a comprehensive combined RPM and CTR programme in recently hospitalised CHF patients. We hypothesize that this intervention has superior effects on physical functional capacity than RPM alone.

**Trial registration:**

Netherlands Trial Registry (NTR) NL9619, registered 21 July 2021.

## Background

Heart failure (HF) is one of the leading causes of hospitalisations, morbidity and mortality worldwide [1, 2]. Heart failure hospitalisation leads to physical and physiological deconditioning, and the readmission rate is high (approximately 22–30%) in the first weeks after discharge [3–6]. Analogous to deconditioning, the frailty rate in the HF population is significant, with prevalence rates over 70% in hospitalised HF patients [7]. Frailty is a clinical syndrome characterised by a decline in physiological reserve and increased vulnerability, initiated by illness and/or ageing. HF, deconditioning and frailty frequently co-exist and are each independently predictors of mortality, disability and hospitalisation [8]. The high hospitalisation, morbidity and mortality rates in HF constitute to an extensive economic burden for healthcare systems.

Cardiac rehabilitation (CR) is an essential part of care for chronic heart failure (CHF) patients to improve health outcomes including quality of life, exercise capacity, and HF-related hospitalisations [9–15]. Current international guidelines recommend multidisciplinary HF management, exercise training (Class I, Level A evidence), and the consideration of home telemonitoring (Class IIb, Level B evidence) for all CHF patients regardless of HF aetiology [16–18]. Despite guideline recommendations, HF patients rarely participate in comprehensive CR programmes and the adherence rate during these programmes is low (approximately only 40% follows the exercise recommendations) [10, 17, 18]. As demonstrated by the HF-Action trial, low adherence rates are independently associated with cardiovascular mortality or HF hospitalisation [19, 20]. Causes of low referral and adherence rates are multifactorial and are determined by system-, professional-, patient-, and disease-related barriers [21, 22]. Examples of system-related barriers are limited CR facilities and lack of reimbursement for CR, and professional-related barriers are lack of endorsement for CR (possibly caused by lack of awareness for the benefits associated with CR participation). Patient-related barriers, such as logistic concerns (e.g. lack of transport), psychological status (e.g. motivation, depression, and anxiety) and socio-economic status, and disease-related barriers as recurrent episodes of decompensated HF and high disability burden in elderly patients are considered to play an even more important role [3, 9, 10, 23–25]. Yet, despite the fact that preliminary analyses showed that benefits of CR are particularly high in this frail elderly population [26], previous CR meta-analyses often excluded recently hospitalised CHF patients and had a relatively low median age (63 years in CR meta-analysis vs. 77 years Danish epidemiological HF-study) [12, 27]. The EJECTION-HF and REHAB-HF trials were the first to show that CR in recently hospitalised CHF patients is feasible and safe. However, a reduction in death or readmission rate could not be demonstrated and adherence to these centre-based CR interventions was poor [4–6]. Remote CR has the potential to help overcome barriers as low adherence and limited involvement of frail elderly in centre-based CR, and will therefore further improve health outcomes.

In the last decade, cardiac telerehabilitation (CTR) has emerged as a safe and effective alternative to centre-based CR for patients with coronary heart disease [28]. Although evidence on the effectiveness of CTR in CHF patients is less abundant, it has the same beneficial effects on quality of life in stable CHF patients as centre-based CR and is superior to no CR on improving functional capacity [29, 30]. Furthermore, in an exploratory study we confirmed that, even in a high-risk elderly population with combined CHF and chronic pulmonary disease, a CTR programme was feasible [31]. Conditions for such a CTR programme to be successful include a patient-centred design including elements for lifestyle and psychosocial guidance, prevention of disease progression, symptom control, and self-management [16]. These elements could be integrated in a non-invasive telemonitoring care pathway (remote patient management; RPM), using wearables, telephone support and a digital platform for early recognition of HF deterioration to reduce the risk for acute decompensated heart failure (ADHF) episodes, recurrent hospitalisations and cardiovascular death [16, 32]. The combination of multidisciplinary HF management, CTR and RPM improves the access to health care (by transferring care to patients’ home environments) and allows care to be adjusted to disease fluctuations while meeting the preferences and needs of individual patients, thereby improving utilisation, adherence, physical functional capacity and health outcomes.

In this study we aim to evaluate the effect of a comprehensive CTR programme combined with RPM in recently hospitalized CHF patients on physical functional capacity. We hypothesize that this intervention has superior effects on the physical functional capacity as compared to RPM alone.

## Methods

### Study design

**Fig. 1:**
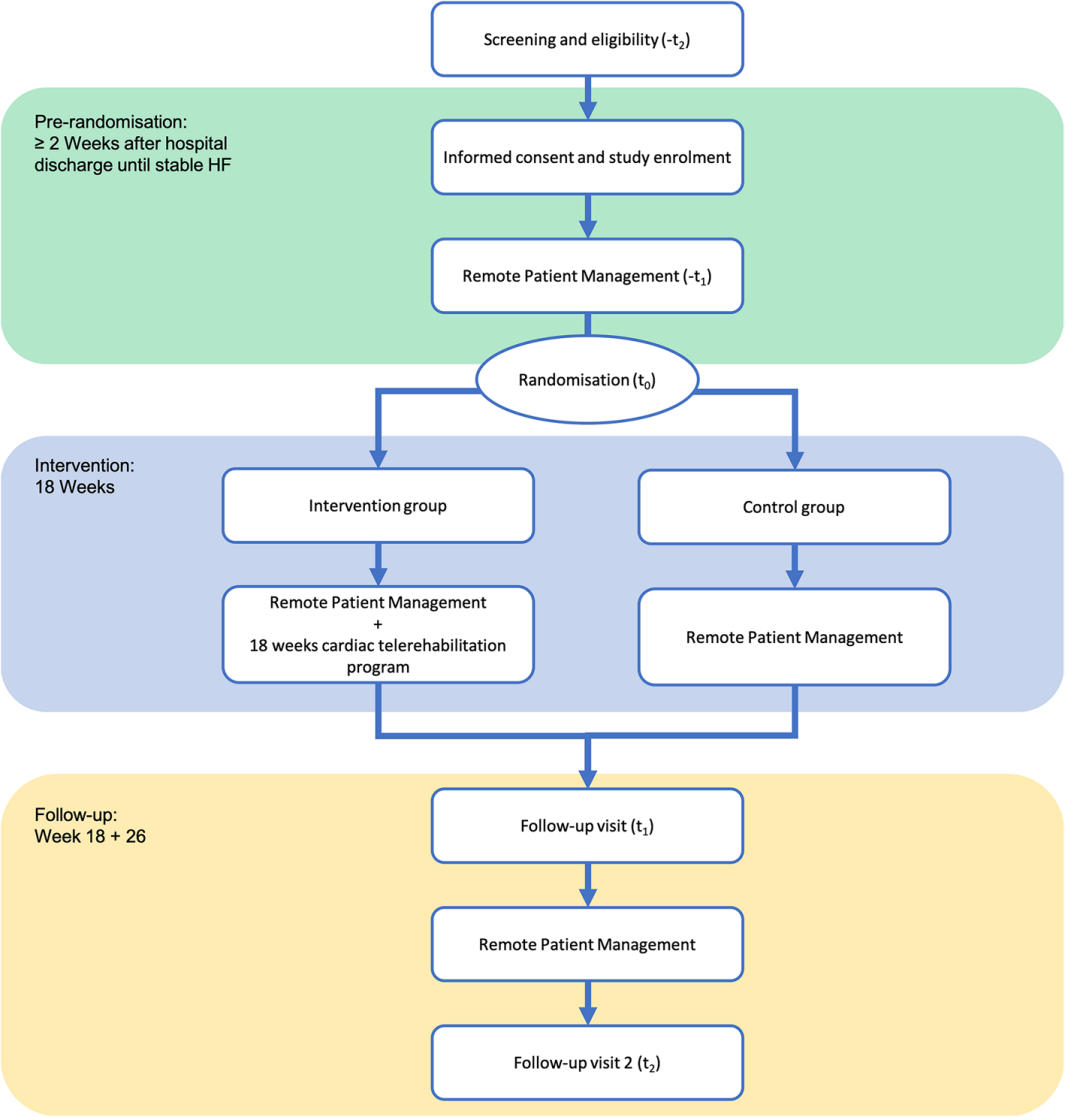
Study design diagram. -t_2_ = at hospitalisation for acute decompensated heart failure, -t_1_ = after hospital discharge until stable heart failure is reached, t_0_ = randomisation and allocation, t_1_ = follow-up visit 1 – 18 weeks after randomisation, t_2_ = follow-up visit 2 – 6 months after randomisation

This study is designed as a randomised controlled trial to evaluate the effect of CTR integrated with an RPM programme compared to RPM without CTR in CHF patients. Participants will be recruited during hospitalisation for ADHF at Máxima Medical Centre, Eindhoven/Veldhoven, and Catharina Hospital, Eindhoven, The Netherlands. Following hospital discharge, all participants will start with RPM and will be followed by their cardiologist and specialised HF nurse for up-titration to optimal medical therapy (OMT). When a stable situation is achieved (defined as OMT and unchanged HF symptoms for at least two weeks), participants will be randomised to either 18 weeks of comprehensive CTR in addition to RPM (intervention group), or RPM without CTR (control group). The period until reaching stable HF is at least 2 weeks after hospital discharge, and will be variable for all participants. All participants will sign informed consent before enrolment. Data will be collected at enrolment (-t_2_), during the pre-intervention period (hospital discharge until stable HF; -t_1_), at randomisation (t_0_), 18 weeks after randomisation (t_1_), and 6 months after randomisation (t_2_) (Fig. 1, Table 1). The study protocol was approved by the local Medical Research Ethics Committee (MREC) of Máxima Medical Centre. The trial is registered at the Netherlands Trial Register (NTR) with registration number Trial NL9619.

**Table 1:**
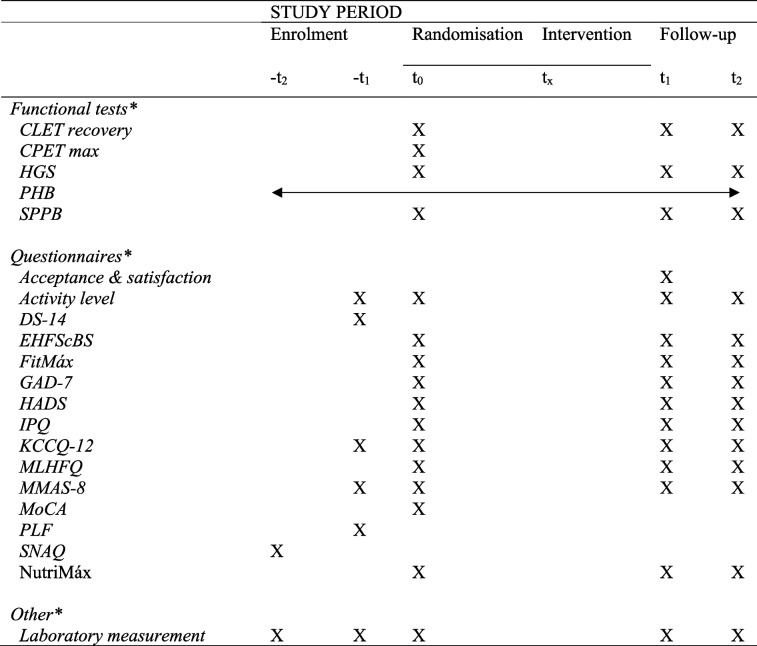
Overview of the assessments during the study period

*-t*_*2*_ at hospitalisation for acute decompensated heart failure, *-t*_*1*_ within 2 weeks after hospital discharge, *t*_*0*_ randomisation and allocation, *t*_*x*_ intervention period, *t*_*1*_ follow-up visit 1 – 18 weeks after the intervention started, *t*_*2*_ follow-up visit 2 – 6 months after the intervention started

*Functional test*: CLET recovery = recovery of the constant-load exercise test, CPET max = maximal cardiopulmonary exercise test, HGS = Handgrip Strength test, PHB = wrist worn device ‘Philips Health Band’, SPPB = Short Physical Performance Battery

*Questionnaire*: Acceptance & satisfaction = questionnaire for acceptance and satisfaction of the CTR programme and digital platform, Activity level = subjective activity level question, DS-14 = Type D personality scale, EHFScBS = European Heart Failure Self-Care Behavior Scale, FitMáx = FitMáx©-questionnaire, GAD-7 = Generalised Anxiety Disorder assessment, HADS = Hospital Anxiety and Depression Scale, IPQ = Illness Perception Questionnaire, KCCQ-12 = Kansas City Cardiomyopathy Questionnaire, MLHFQ = Minnesota Living with Heart Failure Questionnaire, MMAS-8 = adjusted Morisky Medication Adherence Scale, MoCA = Montreal Cognitive Assessment, NutriMáx = nutrition questionnaire based on the national nutrition guideline from the Dutch Health Council, PLF = Premorbid Lifestyle questionnaire, PHQ-9 = Patient Health Questionnaire, SNAQ = Short Nutritional Assessment Questionnaire

*Other*: Laboratory measurements (e.g. NTproBNP, GDF15)

^a^All objectives will be assessed in both groups

### Study population

All patients currently hospitalised for ADHF will be screened for study participation according to the in- and exclusion criteria (Table 2). Patients who are interested will be enrolled before discharge after signing informed consent.

**Table 2:** Inclusion and exclusion criteria

Inclusion criteria:
1) Aged ≥ 18 years
2) Diagnosed with congestive heart failure
3) Hospitalised primarily for acute decompensated heart failure (ADHF) at the time of inclusion
4) Sufficient digital literacy, or caretaker with digital literacy
5) Able to speak and read the Dutch Language
Exclusion criteria:
1) Unable to understand the purpose and procedures of the study
2) Unable to walk a distance of 4 m independently (walking aids are allowed)
3) Cardiac rehabilitation programme followed in the previous 12 months
4) No internet connection
5) Untreated life-threatening cardiac arrhythmia
6) Early phase after acute coronary syndrome (latest 3 months)
7) Uncontrolled hypertension
8) Advanced atrioventricular block
9) Severe aortic stenosis
10) Up-coming major (cardiac) surgery in 3 months

### Randomisation, blinding and treatment allocation

Participants are randomly allocated to the intervention or control group (1:1) after reaching stable HF and completing the first measurements. Participants will be replaced when discontinuing from study protocol before randomisation. Randomisation is performed by the investigator using a computerised randomisation system in the web-based database software Castor EDC (Castor Electronic Data Capture, Ciwit BV, Amsterdam, The Netherlands). The computer system uses variable block sizes (2,4) stratified for age (< 75 years or ≥ 75 years) and left ventricular ejection fraction (LVEF ≤ 40%, LVEF 41 – 49% or LVEF ≥ 50%) for randomisation. Overall, patients with HF are elderly with an average age of 75.2 years in Western Countries as presented by Norhammar et al. [33]. The participants, investigator, and other medical professionals will not be blinded for the treatment allocation due to the nature of the intervention. For the assessment of the functional tests (Short Physical Performance Battery and Handgrip Strength test) a blinded investigator will be involved.

### Remote patient management

Directly after hospital discharge, study participants start with remote patient management (RPM). RPM, also known as telemonitoring, allows health care providers to closely review patient-generated health data, interact with the patient, and initiate clinical treatment if needed [34]. RPM is associated with reduced mortality, and admission rate [32]. Study participants are asked to daily measure their vital parameters (e.g., heart rate, blood pressure) and weight with medical devices (iHealth Track Blood Pressure monitor, iHealth Lina scale) and manually entry the data on the digital monitoring platform (Mibida BV). In addition to the vital parameters, participants are instructed to answer 5 short HF related questions on the platform (e.g., ‘Do you experience shortness of breath?’). Alerts will be generated by the platform based on predefined and individualised reference values. A specialised cardiac nurse will review the patient-generated health data and alerts during the weekdays, and a cardiac care nurse from the Coronary Care Unit / Cardiology Ward will review the data during evenings and weekends. The patient is instructed to contact the nurse by telephone between defined hours when an alert is generated; for urgent matters the medical professionals are available 24/7. Besides daily monitoring to recognise early clinical decompensation, the specialised nurse and cardiologist are responsible for the up titration to optimal medical therapy (OMT) according to ESC guidelines [16]. In a daily meeting with the cardiologist, the nurse will discuss patients with clinically relevant alerts, patients for medication up titration, and patients for remote routine follow-up (2 weeks, and 6 weeks after hospital discharge, and every 3 months). The team will decide what action is needed (e.g. increase in diuretics because of weight gain and oedema). The specialised nurse and cardiologist are primarily responsible for achieving OMT after hospital discharge. All participants will continue with RPM during the entire study period.

### Intervention group: cardiac telerehabilitation

This multidisciplinary 18-weeks CTR programme includes interventions on physical function and activity, diet, and mental health. Physical, nutritional and psychological goals will be assessed by the specialised nurse at the CTR intake procedure. After the general intake, the patient will be referred for an intake with the physical and occupational therapist, dietician, and psychologist.

#### Physical training

Patients in the intervention group start with a (45 min) combined in-hospital intake assessment with the physical and occupational therapist. They will assess the current physical and independence status, the limitations, and discuss personal goals following the latest Dutch CR guideline [35]. During this first assessment, the therapist and patient will determine the focus of the following consults and determine the distribution between occupational and physical follow-up. After the first assessment, the first 3 training sessions will be in-hospital and take 45–60 min each, followed by 2 live-video training sessions of 45 min each, and weekly video coaching sessions which last 20 min with occupational or physical therapist. There will be a multidisciplinary evaluation in week 10 with the occupational and physical therapist. The frequency of the coaching sessions after week 10 will be adjusted in response to the evaluation, with the expectation that 2–4 more sessions are needed in the following 7 weeks. After 18 weeks, there will be a final remote assessment with both therapists.

#### Nutrition intervention

Patients in the intervention group start with an initial video assessment with a dietician to assess and discuss dietary pattern, (unintentional) weight loss, malnutrition, and adherence to sodium and fluid restriction. Before the first consultation, the patients will fill in the NutriMáx-questionnaire based on the nutrition guideline from the Dutch Health Council for providing insight in to the actual nutritional behaviour [36]. Personalised nutrition goals will be discussed by the dietician and patient with specific attention for sodium and fluid restrictions. The nutrition behaviour will be followed during the intervention period using a chatbot, which is a web-based/mobile-based conversational dietary assessment tool used for monitoring daily dietary behaviour. There will be 3 individual video consultations with the dietician, and one group-based video consultation with other CTR participants and dietician. Before the final assessment the NutriMáx will be re-assessed.

#### Psychological intervention

Patients in the intervention group will have a video-based intake assessment with a psychologist for the screening of anxiety and depression symptoms, and the coping strategies used for managing their illness and health. In addition to this assessment, information about psychological status will be obtained from questionnaires (e.g. HADS, GAD-7, PHQ-9, DS-14). The patient and psychologist will determine whether follow-up consultation is needed, and in what form.

#### Digital platform

The study intervention will be performed using a secured, personalised, and patient-centred digital platform (‘My Flow Coach’, Mibida BV, Eindhoven, The Netherlands). The platform is used for daily monitoring of the RPM programme, and visualising the data from the wrist-worn device (Philips Health Band; PHB, Philips Electronics Nederland B.V., Eindhoven, The Netherlands) directly after hospital discharge. The platform has features that enable patients to:

- Register and evaluate vital parameters, and HF related complaints for daily monitoring
- Register and evaluate physical, nutritional, and mental health rehabilitation goals (intervention group)
- Register treatment modules (intervention group)
- Upload and review data from the wrist-worn device (e.g. heart rate, steps, active minutes, energy expenditure)
- Perform video- and chat consultations with health care providers
- Provide relevant caregivers (e.g. cardiologist, specialised cardiac nurse, physical therapist, occupational therapist, dietician, psychologist, investigator) access to relevant clinical data.

### Wrist-worn device

All study participants will receive a wrist-worn device (PHB) at hospital discharge for continuous data collection (e.g. activity counts, heart rate, respiration rate, total energy expenditure, active energy expenditure, steps, and sleep). The PHB provides continues health tracking by measuring the movement and physiological parameters by photoplethysmography technology (PPG). The measurements will be transferred to an application on their mobile phone or tablet via Bluetooth, and to the Philips Actigraphy Server System (PASS). The data will be collected and saved using a study ID (identification) code, and is only accessible by the research team. Participants get access to their raw data in the mobile application and digital platform. They will be asked to wear the wrist-worn device preferably 24 h a day, but at least during every exercise moment. The data will be collected from hospital discharge until 6 months after randomisation.

### Chatbot

**Fig. 2:**
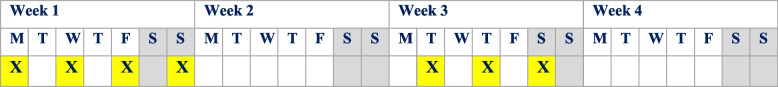
Chatbot schedule – repeated every 4 weeks. Weekdays – Monday to Sunday. X = measuring day

The chatbot application (Mibida BV) will monitor the daily dietary behaviour of the intervention group during the intervention period. The patients will regularly receive multiple choice questions from the chatbot about their intake (e.g. ‘Goodmorning, what did you eat this morning?’) (Fig. 2; Chatbot schedule). The chatbot has the intention to trigger ‘nudging’ based on the Nudge Theory [37], which refers to strategically changing the environment to anticipate on altering peoples’ behaviour without forbidding any options. The chatbot is accessible with an application on a mobile phone or tablet for the intervention group during the intervention period. The data entered by the patients will be visible for the dietician on the digital platform.

### Control group

Both the control and intervention group will continue to use the RPM programme during the study period, given this is part of regular care, and outpatient appointments with the cardiologist and specialised HF nurse will be planned when needed.

### Outcome measures

The primary outcome measure is physical functional capacity at randomisation, 18 weeks and 6 months after randomisation. Secondary outcome measures are recovery after submaximal exercise, maximal exercise capacity, subjective health status and quality of life, personality and behaviour aspects, nutrition behaviour, compliance and acceptance to the intervention, fluctuation of congestive HF biomarkers, and readmission rate.

### Physical functional capacity

Physical functional capacity is assessed with the Short Physical Performance Battery (SPPB) functional test. The SPPB is an objective assessment tool to evaluate the lower extremity function in older persons to reflect the physical self-reliance. It is described as a screening tool to detect frailty [38], and a predictor of major adverse health-related events (e.g. disability, hospitalisation and mortality) in elderly patients [39–41]. The test consists of three parts: gait speed, standing balance and time to rise from a chair. Each test is scored out of 4 points; 0 corresponds with not able to perform the test, and 4 with best performance. A maximum of 12 points can be scored, 0–3 corresponding with severe physical limitation, 4–9 high risk, and 9–12 low risk for developing new physical limitations.

### Maximal exercise capacity and recovery after submaximal exercise

All patients will start the intervention period with a symptom limited maximal Cardiopulmonary Exercise Test (CPET) on a cycle ergometer (Lode Corival, Groningen, The Netherlands) using an individualised ramp protocol aiming of a total test duration of 8–12 min. CPET will be used to determine the maximal exercise capacity and the peak workload, to support the prescription of a tailored exercise rehabilitation programme [42, 43]. The effect of the intervention on maximal exercise capacity, defined as VO_2peak_, will be evaluated with the validated FitMáx©-questionnaire at randomisation, and follow-up [44]. Furthermore, recovery of O_2_ kinetics (τ-rec) after submaximal exercise will be assessed with a Constant-Load Exercise Test (CLET) at 50% of the peak workload at randomisation and follow-up. The CLET includes 2 min of rest, 2 min of unloaded pedalling, 6 min at 50% of the maximum workload, and a resting period of at least 5 min until reaching or approaching the VO_2_ baseline value. Submaximal oxygen uptake kinetics are found to be equally related to functional mobility in elderly and HF patients as VO_2peak_ [45]. CHF patients consume oxygen at a higher level to their peak oxygen uptake than healthy subjects during activities of daily life (ADL), Spruit et al. found a VO_2_ of 38–52% of the peak VO_2_ during ADL [46]. Therefore, CLET at 50% of the peak workload is expected to be indicative of ADL activities, and better tolerated and more representative for changes in physical capacity than maximal exercise [47].

### Frailty risk screening

Frailty has been defined as a clinical syndrome with declines in multiple physiological systems associated with increased vulnerability to stressors related to adverse outcomes, such as disability, falls, hospitalisation, and mortality [48]. Although an universal consensus about an appropriate and accessible screening tool is lacking, the prominent domains are found in the Vigorito frailty assessment tool [49]. In this study, a global frailty screening will be made based on this Vigorito frailty assessment tool [50] using the following domains:

1. Physical activity and function; evaluated with SPPB and Handgrip Strength test (HGS) at randomisation, and follow-up;
2. Malnutrition; evaluated with SNAQ (Short Nutritional Assessment Questionnaire) for malnutrition screening at inclusion;
3. Cognitive impairment; evaluated with MoCA (Montreal Cognitive Assessment) at randomisation;
4. Comorbidities and medication use; evaluated with the number of medications used at inclusion (hospital discharge);
5. Physiological and social status; evaluated with the depression and anxiety screening questionnaires HADS (Hospital Anxiety and Depression Scale), GAD-7 (Generalised Anxiety Disorder assessment) and PHQ-9 (Patient Health Questionnaire) at randomisation, and follow-up.

### Subjective health status and quality of life

Health related quality of life (HRQoL) is evaluated with the KCCQ-12 (Kansas City Cardiomyopathy Questionnaire), and MLHFQ (Minnesota Living with Heart Failure Questionnaire). KCCQ-12 is the shorter version of the self-administered KCCQ, and is assessed to measure patients’ perception on their health status. It includes the frequency of HF symptoms, physical and social limitations, and quality of life (QoL) impairment as a result of HF within a 2-week recall period. The MLHFQ is a 21-item, self-administered instrument developed to independently measure the effect of HF on patients’ lives (in the physical, socio-economic and emotional/physiological domain) over the previous 4 weeks [51]. The KCCQ and MLHFQ are both reliable and validated questionnaires responsive to clinical change, however the KCCQ is more strongly correlated with functional status parameters, and MLHFQ more responsive to improvement in physical functional capacity (6MWT) [52, 53].

### Personality and behaviour aspects

Self-care behaviour and personality characteristics are evaluated with questionnaires to evaluate its influence on the primary outcome. Personality is evaluated with the DS-14 (Type D personality scale), and premorbid behaviour with the PLF (Premorbid Lifestyle questionnaire). Illness perception and self-care behaviour are evaluated using the IPQ (Illness Perception Questionnaire) and EHFScBS (European Heart Failure Self-Care Behavior Scale).

### Nutrition behaviour

Nutrition behaviour is assessed with the NutriMáx-questionnaire based on the Dutch dietary guideline of the Dutch Health Council [36]. NutriMáx consists of 18 questions that will provide an overview of the nutrition behaviour based on the 15 nutrition categories listed in the national nutrition guideline. The total score is scaled from 0 – 32, and represents the adaptation to the guideline. A score of 28 – 32 represents good adaptation, 20 – 27 moderate adaptation, and 19 or below poor adaptation.

### Compliance and physical activity

The data collected by the PHB and stored at PASS is used to determine the compliance and physical activity. Compliance will be evaluated in terms of: (i) the time of wearing the wrist-worn device, (ii) and achievement of the personalised physical goals set by the therapist for the rehabilitation group. Physical activity will be evaluated during the different study phases by change in active and total energy expenditure, and step counts in both groups. Medication adherence and subjective activity level is evaluated with the MMAS-8 (Morisky Medication Adherence Scale) and self-constructed 1-question activity questionnaire.

### Acceptance of the intervention and platform

Satisfaction and acceptance of the CTR programme in general, the chatbot application, and digital platform is assessed using a questionnaire based on the 5-point Likert scale. The control group will only evaluate the satisfaction and acceptance of the digital platform.

### Readmission rate and other adverse events

The readmission rate and other adverse events are assessed during the study period in both groups. Readmission is defined as a hospitalisation for at least 24 h. We will differentiate between HF related causes, other cardiovascular causes, and non-cardiovascular causes of readmission. Other adverse events that will be reported are: 1) acute decompensation without hospital admission, 2) myocardial infarction, 3) emergency room visit without hospitalisation, 4) (cardiac) surgery, 5) admission to a nursing home or rehabilitation centre, 6) (cardiovascular) death, and 7) adverse events that might be related to the intervention.

### Heart failure biomarkers

HF related biomarkers (NTproBNP and GDF15) are measured to evaluate the effect of CTR on fluctuation of these biomarkers, and the predictive value of these markers on physical functional capacity, readmission, and mortality. N-terminal prohormone of BNP (NTproBNP) is a reliable gold standard diagnostic biomarker in HF, and has high prognostic accuracy for death and HF hospitalisation [54]. Growth Differentiation Factor 15 (GDF15) is a less known biomarker, although multiple studies have provided evidence of its prognostic value in CHF patients [55, 56]. These biomarkers are determined at 6 different moments in the study: (I) the first day of the initial hospital admission, (II) at hospital discharge, (III) 1–2 weeks after hospital discharge, (IV) when ‘stable HF’ is reached, (V) 18 weeks after randomisation, and (VI) 6 months after randomisation.

### Statistical analysis

All analyses will be performed according the intention-to-treat principle. Descriptive statistics will be used to present demographic and baseline characteristics. Between- group differences in the primary and secondary endpoints will be analysed by the unpaired T-test for continuous variables and by a chi squared test for categorical variables. A paired T-test will be used to evaluate within-group differences for the primary endpoint.

### Sample size calculation

The sample size calculation is based on the primary outcome measurement – physical functional capacity assessed by the SPPB. Assuming that the study population in general corresponds with lower performance (SPPB score ≤ 9), and that CTR will result in an increase of 1.6 points (SD 2.17) as described by Rengo et al., 64 patients are needed to achieve a statistical power of 0.80 [57]. Previous research found a subjective and quantitative better physical functional capacity with an increase of 0.4 – 1.5 points [58].

## Discussion

The Tele-ADHF trial is the first prospective randomised controlled trial designed to evaluate the effect of a comprehensive CTR programme integrated with RPM on the functional capacity of recently hospitalised CHF patients. Unlike most previous studies, the current CTR programme starts after hospital discharge, thereby targeting the high-risk period in which functional status and quality of life are low and the risk of clinical deterioration is high. By performing randomisation for CTR or usual care after reaching stable HF and OMT, the negative influence of HF instability, medication adjustment and side effects on the adherence to CTR is expected to be limited. Another unique feature of the current study is that frail elderly patients are not excluded. Although previous studies did not include frail patients due to their poor prognosis [59], recent studies showed significantly greater CR treatment success in frail CHF patients, and significantly more reduction in all-cause hospitalisations than in non-frail CHF patients [60].

The primary outcome measurement, physical functional capacity assessed with the SPPB, has better concurrent validity when compared to other measures of frailty and gives a more comprehensive measure of the physical performance when compared to the more frequently used 6MWT (6-Min Walk Test) [61]. SPPB is associated with frailty, disability, hospital admission and mortality, however its prognostic value is not inferior to the 6MWT. In contrast to the 6MWT, the SPPB is more suitable for frailty assessment in elderly by giving separate information on balance, gait speed and strength. Readmission is considered a secondary outcome measurement due to the limited study population size. Frailty screening is essential for elderly (CHF) patients to provide appropriate care and prevent (re)admissions.

This study describes a comprehensive CTR programme that includes the essential components for secondary prevention in HF as mentioned in the latest ESC guidelines; multidisciplinary team management, lifestyle advice, exercise training, follow-up, and monitoring [16]. Most previous CR and CTR studies have focused only the exercise intervention, and did not involve an occupational therapist, dietician and psychologist [12]. Anxiety and depression are common in HF patients, affecting approximately 20% of all HF patients [62]. As anxiety and depression lead to lower CR adherence and social isolation, psychological intervention should be included in CTR to reduce depression and anxiety symptoms, and to improve social functioning and QoL [9, 62]. In addition, fluid restriction and limitation of salt intake may improve HF symptoms, and should be discussed by a dedicated dietician [9]. This 18-week CTR programme, 6 weeks more than nationally recommended, consists of regular, mainly home-based follow-up consults, and the option for additional consults depending on the patients’ need. The extended rehabilitation period will help patients to further adopt and maintain lifestyle changes, and gives more opportunities on providing individualized care. The addition of RPM to the CTR programme makes it possible to closely monitor fluctuation in complaints and vital parameters, and intervene when needed. So, a comprehensive CTR programme addressing multiple domains is considered to be more beneficial than an unidimensional approach on improving patient outcomes and quality of life by providing tailored and individualized care.

This study further distinguishes itself from other CTR studies by the use of multiple innovative technologies (wrist-worn device, chatbot and (group)video consultation) linked to one digital platform. The digital platform is used for multiple purposes; telemonitoring, training evaluation, communication, and generating alerts. The design of the platform stimulates the self-management behaviour of participants by displaying their CR goals and daily progress. The wrist-worn device not only allows the therapist to give tailored advice on the intensity, duration, and frequency of the exercise, but the 24/7 monitoring allows the therapist to give advice on the sedentary behaviour, moments of rest, and all day (household) activities as well. Furthermore, the uses of an application for food tracking and promoting self-awareness in an CTR programme has not been described before [63, 64]. Individual and group video consultation is added to the intervention as well to promote personal contact and social interaction.

### Limitations

This study has a number of limitations. First, with the use of a digital intervention, participants with insufficient digital literacy are excluded which may lead to a selection bias. However, this effect is expected to be limited through the involvement of caretakers and home care organisations at inclusions and follow-up. Furthermore, one of our previous studies with a similar intervention has already shown feasibility of such a digital intervention in elderly CHF patients [31] and improvements have been made to the programme in response to patient experiences and feedback on this previous intervention. Finally, elderly people have significantly increased their digital skills over the past decade, making it highly likely that they will be able to use the digital intervention, and that future implementation of this programme will be successful [65].

Second, due to the comprehensiveness of this CTR intervention, participation may be too demanding for the patients in the intervention group. In the design of the care pathway, this potential burden has been dealt with by: 1) individualising the program and consultation frequency to the patient’s needs, 2) involving caretakers, 3) regularly monitoring the patient’s progress and adjust if necessary, 4) using video consultation to limit clinical appointments, and 5) adjusting the platform with the feedback from the previous study.

A final limitation is that the control group will not receive CTR in the first period after hospital discharge. However, the referral rate in the CHF population is still generally low, especially for those who were admitted recently or are classified as frail. In this study, all participants will be offered CR eventually; the intervention group during the study period and the control group after finishing the follow-up period. Furthermore, this study is expected to increase awareness on benefits of CR and will therefore hopefully improve the referral rate.

## Conclusion

The Tele-ADHF study is the first study to evaluate the effects of an innovative integrated care pathway combining CTR and RPM for recently hospitalised CHF patients. It will provide new insights in the optimisation of follow-up and care for CHF patients, with the ultimate goal being to reduce hospitalisations, morbidity and mortality in this high risk patient group.

## Data Availability

Not applicable.

## Abbreviations

6MWT: Six minute walking test
ADHF: Acute decompensated heart failure
ADL: Activities of daily living
CHF: Chronic heart failure
CLET: Constant-load exercise test
CPET: Cardiopulmonary exercise test
CR: Cardiac rehabilitation
CTR: Cardiac telerehabilitation
DS-14: Type D personality scale
EDC: Electronic data capture
EHFScBS: European Heart Failure Scale-Care Behavior Scale
ESC: European Society of Cardiology
GAD-7: Generalised Anxiety Disorder Assessment
GDF15: Growth differentiation factor 15
HADS: Hospital Anxiety and Depression Scale
HF: Heart failure
HGS: Handgrip Strength test
HRQoL: Health related quality of life
IPQ: Illness Perception Questionnaire
KCCQ-12: Kansas City Cardiomyopathy Questionnaire-12
MLHFQ: Minnesota Living with Heart Failure Questionnaire
MMAS-8: Morisky Medication Adherence Scale-8
MoCA: Montreal Cognitive Assessment
MREC: Medical Research Ethics Committee
NTproBNP: N-terminal pro b-type natriuretic peptide
NTR: Netherlands Trial Register
OMT: Optimal medical therapy
PASS: Philips Actigraphy Server System
PHB: Wrist worn device ‘Philips Health Band’
PLF: Premorbid Lifestyle questionnaire
PHQ-9: Patient Health Questionnaire-9
PPG: Photoplethysmography
QoL: Quality of life
RPM: Remote patient management
SNAQ: Short Nutritional Assessment Questionnaire
SPPB: Short Physical Performance Battery
τ-rec: Recovery of O_2_ kinetics
